# Epidemiological analysis of doping offences in the professional tennis circuit

**DOI:** 10.1186/1745-6673-5-30

**Published:** 2010-12-15

**Authors:** Javier Maquirriain

**Affiliations:** 1 High Performance National Sports Center, Buenos Aires, Argentina; 2 Argentine Tennis Association, Buenos Aires, Argentina

## Abstract

**Introduction:**

Tennis is a professional sport under a strict anti-doping control. However, since the first violation of the code, the positive cases have not been statistically studied. The objective of this study was to analyze doping offences in the international professional tennis circuit.

**Methods:**

All offences to the Doping Code committed by tennis players during 2003-2009 were collected from the ITF official webpage, registered and analyzed.

**Results:**

An average of 1905.7 (±174.5) samples was obtained per year. Fifty-two doping offences were reported and the overall incidence of positive doping samples accounted for 0.38% and 7.4 (±4.1) cases/year. Male players showed higher incidence doping offences than females (p = 0.0004). The incidence in wheelchair players was higher than in non-handicapped subjects (p = 0.0001)

Banned substance distribution showed: *stimulants *32.69%, *cannabis *23.07%; *anabolic *11.53%, *diuretics and masking agents *11.53, *β2-agonists *9.61%; *corticosteroids *3.84%, *others *3.84%. The overall incidence of *'social drugs' (cocaine, cannabis) *was 36.53%. All EPO and blood samples were normal, while the incidence of *'out-of-competition' *offences was 0.12%. The lower incidence of doping was found in Grand Slams tournaments.

**Conclusions:**

The incidence of positive doping samples among professional tennis players is quite low supporting the assumption that there is no evidence of systematic doping in Tennis. *"Social drugs" *misuse constitutes the main problem of doping in tennis. Male and wheelchair tennis players showed higher risk of infringing the doping code than their females and non-handicapped counterparts. Findings of this study should help to determine the direction of the ongoing strategy in the fight against doping in Tennis.

## Introduction

Tennis is one of the most popular sports throughout the world. The number of professional players, both male and female, is continually increasing each year. At present, there are 1794 male players who have ATP* singles ranking [1] and 1106 women players who have WTA** ranking [2]. The professional tennis circuit manages its doping control through the Tennis Anti-Doping Programme (TADP) which is an international comprehensive drug-testing system that applies to all players who hold an Associate Tennis Professionals (ATP) or Women Tennis Association (WTA) ranking, or who are competing at tournaments sanctioned by the International Tennis Federation (ITF), ATP, and WTA Tour. This includes men's and women's tour events, Grand Slams, Fed Cup, Davis Cup, wheelchair and junior tournaments. The goals of the TADP are to maintain the integrity of tennis and protect the health and rights of all tennis players [3]. The TADP maintains a common set of rules and procedures that apply across all levels of tennis. Players are tested for banned substances in accordance with the guidelines of the World Anti-Doping Agency (WADA) Code [4].

Drug testing conducted by the Men's Tennis Council began in the late 1980 s and focused on recreational drugs. When the ATP Tour was formed in 1990, the governing body of the men's professional tennis circuit extended the testing to include performance-enhancing drugs. The TADP began in 1993, with each of the three bodies (ITF, ATP and WTA Tour) managing testing at their own events, and dealing with any cases arising from their tournaments. The TADP also conducted *'out of competition' *testing. Since 2007, the ITF has managed, administrated and enforced the TADP at all tennis events sanctioned. These include the Grand Slams, ATP circuit, Sony Ericsson WTA Tour, Davis Cup and Fed Cup, Challenger events, ITF Pro Circuit, and ITF Junior and Wheelchair events.

Since the first anti-doping violation infringed by a 23-year old Spanish player in a Challenger tournament in 1996, only few short reports were published regarding the positive cases [5,6].

The objective of this study was to analyze doping offences in the international professional tennis circuit.

*Association of Tennis Professional, ** Women Tennis Association.

## Methods

The 2009 WADA Code [4] defines 'doping' as the occurrence of one or more of the anti-doping rule violations, such as: 1) presence of a 'prohibited substance', or its metabolites or markers in an athlete's sample; 2) use or attempted use by an athlete of a 'prohibited substance' or a 'prohibited method'; 3) refusing or failing without compelling justification to submit to sample collection; 4) violation of applicable requirements regarding athlete availability for 'out-of-competition' testing; 5) tampering or attempted tampering with any part of doping control; 6) possession of 'prohibited substances' and 'prohibited methods'; 7) trafficking or attempted trafficking in any 'prohibited substance' or 'prohibited method'; 8) administration or attempted administration to any athlete 'in-competition' of any 'prohibited method' or 'prohibited substance', or administration or attempted administration to any athlete 'out-of-competition' of any 'prohibited method' or any 'prohibited substance' that is prohibited 'out-of-competition', or assisting, encouraging, aiding, abetting, covering up or any other type of complicity involving an anti-doping rule violation or any attempted anti-doping rule violation.

The ITF has published the complete list of anti-doping offences between 2003 and 2009 [3]. According to the doping definition, all offences to the WADA Code committed by tennis players that period were collected from the ITF official webpage, registered and analyzed by substance, gender, nationality, and type of tournament.

Descriptive statistics were obtained and chi-square tests were performed for comparing data from different groups within samples (alfa 0.05; beta 0.2). Spearman test was used for correlation analysis. (Statistical package: *Statistica for Windows, Statsoft^® ^Tulsa, Oklahoma, USA)*.

## Results

An average of 1905.7 ± 174.5 doping samples was obtained per year: urine = 1725.1 ± 183.4; blood = 180.5 ± 34.3 (Table 1). Fifty-two doping violations were reported during 2003-2009 and the overall incidence of positive doping samples accounted for 0.38% (52/13340). The annual rate was 7.42 ± 4.11 (range 2-14). Correlation analysis of doping offences and number of samples obtained per calendar year failed to show statistical significance (p = 0.58, Spearman R: -0.2522).

**Table 1:** Descriptive data of control samples and doping offences in the professional tennis circuit in the 2003-2009 period.

Year	N° offences*	Samples* (total)	Urine	Blood	In-Comp	Out-Comp
**2009**	7	2126	1991	135	1972	154

**2008**	10	2018	1861	157	1927	91

**2007**	3	2028	1833	195	1871	157

**2006**	9	1733	1538	195	1583	150

**2005**	14	1744	1589	155	1654	90

**2004**	7	1698	1509	189	1620	78

**2003**	2	1993	1755	238	1912	81

**Total**	52	13340	12076	1264	12539	801

**Average/year**	7.4 ± 4.1	1905.7 ± 174.5	1725.1 ± 183.4	180.5 ± 34.3	1791.2 ± 165.1	114.4 ± 37.0

'In-Comp': in competition testing; 'Out-Comp': out of competition testing.

* Correlation analysis of doping offences and number of samples obtained per calendar year failed to show statistical significance (p = 0.58, Spearman R: -0.2522).

Relative gender frequency of doping cases in professional tennis players was 86.53% (45/52) male and 13.46% female (7/52). Moreover, male tennis players showed significant higher incidence of performing a doping offence than females (45 offences/8373 samples, and 7/4967, respectively; chi-square = 12.6, p = 0.0004) (Table 2). Male tennis players who committed doping violations were older than their female counterparts (27.35 ± 4.51 and 24.14 ± 4.59 years, respectively; p = 0.09, *t*-test for independent samples).

**Table 2:** Analysis of gender differences within doping infractions in the professional tennis circuit during the 2003-2009 period.

	Female Players	Male Players	*p *value
**Total Samples**	4967	8373	

**Sample Average/year**	709.5 ± 178.0	1196.1 ± 91.3	

**Total Doping Offences**	7	45	

**Doping Offences/year**	1.0	6.4	

**Incidence Offences/sample (%)**	0.14	0.53	0.0004

**Age Average of Offenders**	24.1 ± 4.5	27.3 ± 4.5	0.09

Prohibited substances founded in the doping controls of tennis players during the 2003-2009 period showed the following distribution: *stimulants *(S6) 32.69%, *cannabis *(S8) 23.07%; *anabolic *(S1) 11.53%, *diuretics and masking agents *(S5) 11.53%, *β2-agonists *(S3) 9.61%; *corticosteroids *(S9) 3.84%, *others *3.84% (Table 3). The overall incidence of *'social drugs' (cocaine, cannabis) *accounted of 36.53% (19/52) of all cases and none doping cases where found in neither *'blood samples' *nor erythropoietin (EPO) analysis (0/1264 samples).

**Table 3:** Summary of 'prohibited substances' found in doping controls samples of professional tennis players during 2003-2009 (n = 52).

Prohibited Substance	Relative Distribution	Drugs founded in urine analysis
Stimulants (S6)	32.6%	caffeine, ephedrine, cocaine, pemoline, etilefrine, adrafinil, modafinil, isometheptene, nikethamide, methylhexanamine

Cannabis (S8)	23.07%	THC

Anabolics (S1)	11.53%	clenbuterol, stanozolol, nandrolone

Diuretics & Masking Agents (S5)	11.53%	hydrochlorothiazide, finasteride, amiloride, canrenone

β2-agonists (S3)	9.61%	salbutamol, terbutaline

Corticosteroids (S9)	3.84%	betamethasone, triamcinolone, budesonide

Others Infractions	3.84%	

Note: caffeine and finasteride are not banned by the 2010-WADA Code

The TADP includes both, *"in-competition" *and *"out-of-competition" *tests. The average of *"in-competition" *and *"out-of-competition" *tests obtained per year was 1791.2 ± 165.1 and 114.4 ± 37.0, respectively. The overall incidence of *'out-of-competition' *positive cases was 0.12% (1/52), while the specific incidence account of 0.12% (1/801 *'out-of-competition' *samples).

The four Grand Slams tournaments (Australian Open, Roland Garros, Wimbledon and US Open) are the most prestigious individual competitions in tennis. Most of the doping controls 40.86% (5452/13340) were performed during Grand Slams events and the incidence of positive cases in such tournaments was 0.18% (10/5452) (Table 4). Usually blood tests and EPO tests are performed in Grand Slams events exclusively. The Davis Cup is the largest annual international male team competition in sport, while the Fed Cup is the national women team competition in tennis. In the present study, 3.29% (440/13340) of doping controls were obtained during Davis Cup matches, and 2.66% (355/13340) in Fed Cup matches. All participants in Davis cup and Fed Cup final ties were tested in the 2003-2009 period. The incidence of positive doping cases in Davis Cup male players was 0.68% (3/440); no Code violations were reported in Fed Cup female participants (0/355) (chi-square test 2,43; p = 0.11). The incidence of positive doping cases in Grand Slam tournaments (10/5452) were significantly lower than in Davis Cup matches (3/340), and other professional championships, excluding wheelchair tournaments (34/7612), (p = 0.0082 and p = 0.00103, respectively) (Table 4).

**Table 4:** Analysis of doping infractions in different professional tournaments during the 2003-2009 period.

	Grand Slams	Davis Cup	Fed Cup	Other Tournaments	Total
**Total Samples**	5452	440	355	6817	13340

**Sample Average/year**	778.8	62.8	50.7	973.8	1905.7 ±174.5

**Total Doping Offences**	10	3	0	34	52

**Doping Offences/year**	1.42	0.42	0	4.8	7.4 ± 4.1

**Incidence Offences/sample (%)**	0.18*^b^*	0.68*^a-b^*	0*^a^*	0.4	0.38

*^a ^p *= 0.11

*^b ^p *= 0.0082

The TADP also conducted controls in the professional wheelchair tennis circuit at an annual average of 39.8 analyses (range 21-53). In the present study, 9.61% (5/52) of all offences were committed by handicapped players. Wheelchair tennis players showed a significant higher incidence of doping offences than the non-handicapped players: 1.81% (5 violations/276 doping controls) and 0.35% (47/13064) respectively (chi-square = 14.75, p = 0.0001) (Table 5).

**Table 5:** Analysis of doping infractions in wheel-chair and non-handicapped tennis players during the 2003-2009 period.

	Non-handicapped players	Wheel Chair Players	Total
**Total Samples**	13064	276	13340

**Sample Average/year**	1866.2	39.4	1905.7 ± 174.5

**Total Doping Offences**	47	5	52

**Doping Offences/year**	6.7	0.7	7.4 ± 4.1

**Incidence Offences/sample (%)**	0.35*^a^*	1.81*^a^*	0.38

*^a ^p *= 0.0001

The present study showed that the majority of doping offences were committed by European players (59.6%, 31/52), followed by North Americans (15.3%, 8/52), South Americans (15.3%, 8/52), Oceanians (5.7%, 3/52), Africans (1.9%, 1/52), and Asian players (1.9%, 1/52). The most affected countries were France (7 positive doping violations), USA (n = 6), Spain (n = 5), and Argentine (n = 5).

The average duration of sanctions for doping violations in the professional tennis circuit was 13.09 ± 15.35 months (range 0-96 months).

## Discussion

The main finding of this study was the relative low incidence (0.38%) of positive doping samples among professional tennis players, especially for the true performance enhancing drugs such as anabolic steroids and stimulants supporting the assumption that there is no evidence of systematic doping in Tennis. Tennis showed similar doping incidence to those of other sports under strict anti-doping control. The *Fédération Internationale de Football Association (FIFA) *reported a 0.4% relative incidence of positive samples of more than 20,000 controls per year [7]. The incidence of doping cases in the Olympic Games since the implementation of doping controls (1968-2008) was 0.43% [8]; in Beijing 2008 the incidence of positive cases were 0.19% (9/4470) but most of the offences were due to anabolic consumption [8].

Male competitive tennis players showed significant higher incidence of doping offences than female tennis players. Similar results have been reported in others sports. Male tennis players were noted to have more risk-taking behavior than female players [9].

This study also confirmed that *"social drugs" *constitutes the main problem of doping in tennis. As in most other sports, most of doping violations in tennis are due to cocaine and marijuana positive urine samples; furthermore, its relative incidence seems to be increasing in last years [6]. Substance abuse among adolescents and young adults remains an issue of concern in today's society. In the athletic environment, most problems related with recreational drugs include alcohol, marijuana and cocaine consumption, while other less familiar substances (heroin, gamma hydroxy butirate, etc.) are seldom abused. Several studies have shown an impressive increase in the frequency and quantity of marijuana consumption, essentially in the younger population, with an earlier onset of use [10]. Other articles have shown noticeable differences between substance abuse in athletes and non-athletes. For example, athletes showed significant higher risk-taking behaviour than their non-athletic peer. Athletes in contact and team sports demonstrated higher risk of recreational drug abuse than athletes in non-contact and individual sports like tennis [9,11,12]. Other risk factors include high psychological stress and lack of familiar support. Consequently, regular sports participation does not prevent substance abuse like marijuana. Data gathering from WADA-accredited laboratories show that cannabis is easily the commonest drug leading to positive results in all sports [11]. Cannabis is prohibited in Olympics events since 1989 and in professional tennis since the *ATP tour *signed the WADA code in 2002. We consider that the prohibition of marihuana usage in tennis has provided clear benefits for players. In the daily practice, this rule acts as a true restrain for players because they try to reduce or avoid cannabis consumption in order to be allowed for professional participation under the Anti-Doping Code. Some other players had to retire from professional competitions probably due to their addiction to marihuana.

Alcohol consumption is not prohibited in tennis. However, the dangerous increase in alcohol beverages consumption observed among tennis players of all ages in last years should alert sports physicians due to the intrinsic deleterious effects of ethanol, as well as of its facilitating role for marijuana and cocaine misuse. The WADA, the world governing body in doping, states that *"doping in sport results from a combination of individual, cultural, societal, and physiological factors. Prevention of doping in sport must be based on a clear understanding of the complex nature of the problem and the comprehensive mix of strategies needed to address them successfully" *[4]. Since 2001, WADA has committed 50 million dollars to research in the fight against doping [4]. However, surprisingly none of the 186 projects supported by WADA was related to misuse of social drugs in sports.

Another issue of concern is the increasing number of "non-intentional" doping cases in sports [13,14]. Pluim [5] reported that 67.5% of doping cases in tennis at independent hearings accepted that there was no intent to enhance performance. Nutritional supplements can be a source of positive doping case as some supplements contain prohibited substances without showing this on their label [13]. With the number of false-positive doping cases steadily increasing, we should critically review the products that are on the list of prohibited substances and focus on those that are truly performance-enhancing and damaging to health [5].

The Tennis Anti-Doping Program has been conducting *'out-of-competition' *testing since its creation in 1993. The relative incidence of doping offences *'out-of-competition' *was extremely low (0.12%, 1/801). Only one case of such controls was reported when a 23- year old Spanish player refused to give a sample during a 2005 Challenger tournament in Italy. Recently, two Belgian players were suspended because they failed to provide the whereabouts information properly infringing the *'out-of-competition' *rules.

More than 40% of all doping samples were obtained during Grand Slam tournaments. The lower incidence of doping cases was found during these physically and mentally demanding events. We hypothesize that Grand Slam tournaments are played only by true elite tennis players who are more familiarized with anti-doping rules and less prone to commit code violations.

Wheelchair tennis also has an international tour with currently over 120 events taking place all over the world. To be eligible to compete, a player must have a medically diagnosed permanent mobility related physical disability, which must result in a substantial loss of function in one or both lower extremities. In the present study, wheelchair players showed a significant higher incidence of doping violations than those non-handicapped players. The five cases reported misuse of marijuana (3 cases), cocaine (1 case) and modafinil plus adrafinil (1 case). The education of players, coaches, and medical personnel in contact with wheelchair tennis players must be reinforced in order to protect their health and the integrity of this fast growing sport for handicapped subjects.

We also analyzed the nationality of doping offenders. Most of violations were committed by European tennis players. Europeans were also the more sanctioned athletes in the all the Olympic Games (62%) [8]. The present study showed that players from countries where tennis is more popular providing high number of players to the professional circuit (like France, USA, Spain and Argentine), may be more prone to infringe de Anti-Doping Code.

Despite the fact that the incidence of positive doping cases among tennis players is low in comparison with other sports, a stringent system of doping control is critical to the future in the sport. However, findings of this study should help to determine the nature and direction of the ongoing strategy in the fight against doping in tennis: 1) the overall incidence of doping offences is low; 2) the abuse of EPO and growth hormone (GH) is null; 3) the incidence of positive cases in *'out-of-competition' *testing is null too; 4) there is lack of positive correlation between the number of anti-doping controls and positive cases. According to this scientific evidence, the cost-effectiveness relationship of the TADP should be review, and more financial resources may be redirected to different areas of Sports Medicine.

In summary, this study showed that the incidence of positive doping samples among professional tennis players is quite low supporting the assumption that there is no evidence of systematic doping in Tennis. This study confirmed that *"social drugs" *misuse (marijuana and cocaine) constitutes the main problem of doping in tennis. All *'out of competition'*, EPO and GH analysis were negative. Male and wheelchair tennis players showed higher risk of infringing the doping code than their females and non-handicapped counterparts.

## Competing interests

The authors declare that they have no competing interests.
